# A Survey of Potentially Pathogenic-Incriminated Arthropod Vectors of Health Concern in Botswana

**DOI:** 10.3390/ijerph181910556

**Published:** 2021-10-08

**Authors:** Mmabaledi Buxton, Malebogo Portia Buxton, Honest Machekano, Casper Nyamukondiwa, Ryan John Wasserman

**Affiliations:** 1 Department of Biological Sciences and Biotechnology, Botswana International University of Science and Technology, P/Bag 016, Palapye 10071, Botswana; machekanoh@biust.ac.bw (H.M.); nyamukondiwac@biust.ac.bw (C.N.); ryanwas21@gmail.com (R.J.W.); 2 Department of Sociology, University of Botswana, P/Bag UB 0022, Gaborone 00704, Botswana; mcleb97@hotmail.com; 3 Department of Zoology and Entomology, Rhodes University, Makhanda 6140, South Africa

**Keywords:** community knowledge, perceptions and practices, emerging-reemerging diseases, parasites, public health, vectors, vector control, vector-borne diseases

## Abstract

Arthropod vectors play a crucial role in the transmission of many debilitating infections, causing significant morbidity and mortality globally. Despite the economic significance of arthropods to public health, public knowledge on vector biology, ecology and taxonomic status remains anecdotal and largely unexplored. The present study surveyed knowledge gaps regarding the biology and ecology of arthropod vectors in communities of Botswana, across all districts. Results showed that communities are largely aware of individual arthropod vectors; however, their ‘potential contribution’ in disease transmission in humans, livestock and wildlife could not be fully attested. As such, their knowledge was largely limited with regards to some aspects of vector biology, ecology and control. Communities were strongly concerned about the burden of mosquitoes, cockroaches, flies and ticks, with the least concerns about fleas, bedbugs and lice, although the same communities did not know of specific diseases potentially vectored by these arthropods. Knowledge on arthropod vector control was mainly limited to synthetic chemical pesticides for most respondents, regardless of their location. The limited knowledge on potentially pathogen-incriminated arthropod vectors reported here has large implications for bridging knowledge gaps on the bio-ecology of these vectors countrywide. This is potentially useful in reducing the local burden of associated diseases and preventing the risk of emerging and re-emerging infectious diseases under global change.

## 1. Introduction

Arthropod vectors significantly account for human morbidity and mortality through the surge in vector-borne diseases globally [1,2]. It is estimated that vector-borne diseases account for about one million deaths annually, contributing to ~17% of all infectious diseases worldwide [3]. These arthropod vectors are incriminated mainly with pathogens and parasites (e.g., protozoans, bacteria, fungi, viruses and helminths) [4,5,6,7] that cause various infectious diseases to humans, livestock and wildlife [8,9,10]. Among others, mosquitoes, bedbugs, fleas, cockroaches, flies (e.g., biting midgets, black flies, blow flies, house flies, flesh flies, stable flies, tabanid flies and tsetse flies), lice and some arachnids (e.g., ticks and mites) are important arthropod vectors of global medical and veterinary health concerns [11,12]. The diversity and distribution of arthropod vectors of medical significance may vary in space and time [13]. As such, local surveillance studies are significant in updating practitioners in public health, ecology and biodiversity, as well as policymakers and communities on new vectors and potential parasites as an early warning system [14].

In Botswana, documented literature on a diversity of arthropod vectors is limited. Studies are particularly more skewed towards mosquitoes and ticks, presumably due to the burden of malaria [15,16,17,18] and animal health (e.g., heartwater disease) countrywide [19,20,21,22]. Although studies on the duo have been explored, reports on other arthropods of medical and veterinary concern are scant. As such, there is limited information on community knowledge on arthropod vectors and their associated diseases. Given the lack of this knowledge, societies are impacted differentially by debilitating effects of vector-borne diseases through habitat and vector-host dynamics. Consequently, societal knowledge on crucial aspects of vector control, biology and disease ecology remains key in mitigating such impacts at local and regional scales [23]. Thus, solving this problem through identifying and bridging knowledge gaps on the bio-ecology and control practices of important arthropod vectors across communities countrywide is highly warranted.

Arthropod vector success is facilitated by conducive bio-physical factors [24]. For instance, temperature, precipitation and humidity play a crucial role in arthropod vector key life history traits [25,26]. Further, the current increases in average temperatures with climate change are likely to increase arthropod vector spatial and temporal distribution and abundance dynamics across small- to large-scale patterns [27,28,29]. Pathogen/ parasite incubation and virulence are likely also enhanced by an increasingly favourable warming climate [30,31,32,33]. Similarly, the prevailing biotic environment may also have an effect on arthropod vector abundance [34,35]. For example, changes in host abundance and diversity associated with anthropogenic activities may help promote certain pathogens/parasites and vector species more than others [36].

Human-mediated activities and socio-economic status within communities contribute to the abundance and persistence of arthropod vector burdens and associated direct and indirect implications [34,35]. For instance, provision of suitable refugia across macro and microhabitats [37], proximity to several alternative hosts [38], poor living conditions [39], feeding preferences and host availability [40] are key in attracting and influencing population increases of arthropod vectors. However, in some arthropod vectors (e.g., lice), improved personal hygiene [41,42], enhanced socio-economic livelihood [43] and acquisition of appropriate knowledge [44] remain crucial in suppressing their burden. Consequently, in many urban-to-rural land-use gradients, societies are considerably impacted differentially by debilitating effects of vector-borne disease mainly due to habitat variability, vector-host dynamics and human knowledge on crucial aspects of vector control, biology and disease ecology [23,45].

Arthropod vectors are controlled by several approaches including genetic, chemical, environmental manipulation/management and biological methods or a combination of these [46,47,48]. However, synthetic insecticides have been the most widely used globally [49,50,51]. Although synthetic insecticides have, over decades, played a pivotal role in the control of arthropod disease vectors [52,53], indiscriminate usage has been a common practice of significant concern especially in pest management across the agricultural sector [54,55]. Often, a lack of public education and access to information results in compromised efficacy of synthetic pesticides’ control interventions [55]. There is growing concern worldwide over the commercial use of synthetic pesticides and resistance development for arthropod vectors [17,56,57,58,59]. Similarly, the misuse of domestic synthetic pesticides may further exacerbate and induce insecticide resistance at household level as in keeping with Mougabure-Cueto and Picollo [60]. Although insecticide resistance patterns have not been established on arthropod vectors countrywide, malaria vectors displayed resistance to commonly used synthetic pesticides in the malaria stratified zones [17].

Botswana has a stratified gradient of diverse socio-economic lifestyles and livelihoods of communities across rural-urban landscapes. The association of humans, domestic animals and wildlife is often an important interaction in the country [61,62]. This human-livestock-wildlife sympatry may accelerate the interaction of arthropod vectors and associated parasites’ transmission. However, despite these concerning factors, little is known on a variety of disease arthropod vectors and their associated impacts in local communities. To date, most work on arthropods has been spatially restricted to the malaria endemic districts of Botswana and targeting only anopheline mosquito species [15,17,63,64], while neglecting other vectors and the larger geographic space thereof (although see Bango et al. [16]). Given the skewed research and funding priority, biased towards malaria burden concerns in specific regions and districts (endemic areas), the country lacks a full spectrum of mosquito and other arthropod vectors of health concern that could be emerging or re-emerging in less explored, non-endemic areas. It is also highly likely that local knowledge on disease arthropod vectors is similarly skewed. 

The aim of the present study was, therefore, to assess public knowledge on arthropod vectors (cockroaches [Blattodea], ticks [Acari], lice [Phthiraptera], bedbugs [Hemiptera: Cimicidae], house flies [Diptera: Muscidae], mosquitoes [Diptera: Culicidae] and fleas [Siphonaptera]) given the stratified societal livelihoods, human-domestic animal-wildlife interfaces and diverse landscapes in the country. It was hypothesised that (i) a variety of arthropod vectors would be reported to exist in the communities countrywide, given host diversity and despite lack of documented reports, and that (ii) urban pests (e.g., cockroaches and flies) will be more prevalent in developed locations than rural settlements. It was further hypothesised that (iii) community knowledge on arthropod vectors would be largely limited, and that (iv) knowledge would be skewed toward malaria as a prominent mosquito-borne infectious disease in the country with high media and budget coverage and that (v) the use of chemicals would dominate control of arthropod vectors given their availability nationwide. Such information is useful in informing community awareness programs and sustainable control practices needed for improved public health issues, improved self-protection, services and epidemiological systems. Furthermore, it will help the public health sector in strengthening awareness and crafting educational tools that promote individual and household level knowledge acquisition for improved vector and associated diseases’ control across African communities.

## 2. Materials and Methods

### 2.1. Sampling Technique

**Figure 1:**
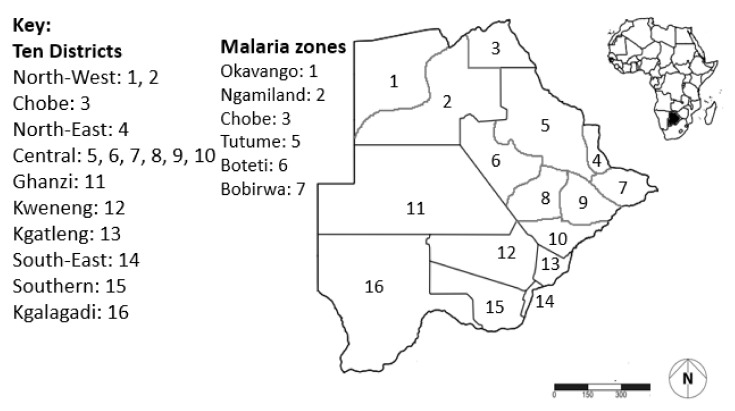
A map of Botswana showing all 10 districts across the country from which respondents were interviewed telephonically. The confirmed malaria cases per 1000 population range from 0 (4, 8, 13, 16), 0–0.1 (10, 12), 0.1–1 (5, 6, 9, 15), 1–10 (2, 3, 7, 11) and 10–50 (1) in keeping with Bango et al. [16] and Kgoroebutswe et al. [70]. The national mosquito vector programme is currently deployed as Main (Indoor Residual Spraying; 1, 2, 3, 5, 6, 7) and supplementary interventions with Long lasting Insecticide treated Nets (1, 2, 3, 5, 6, 7) and piloted Larviciding (5, 6, 7) [70]. The level of knowledge on mosquito bio-ecology and associated diseases has been assessed at 1, 7, 8 and 9 [69,71].

The study was conducted in all 10 districts of Botswana between July and August 2020 (Figure 1). Due to the imposed movement and contact restrictions by COVID-19 protocols locally during this period [65,66], the study was carried out telephonically following protocols of Potter et al. [67]. A telephone directory was used to establish contact with every 10th individual in the contact list from various cities, towns and villages within a district to standardise data. The respondents (*N* = 1048) were from Central; 638,604 (*N* = 109), Chobe; 23,347 (*N* = 105), Ghanzi; 43,095 (*N* = 105), Kgalagadi; 50,752 (*N* = 100), Kgatleng; 91,660 (*N* = 89), Kweneng; 304,549 (*N* = 111), North-East; 159,225 (*N* = 107), North-West; 152,284 (*N* = 107), South-East; 345,613 (*N* = 110) and Southern districts; 215,775 (*N* = 104) (see Figure 1; [68]). A structured questionnaire was used to assess the knowledge of the respondents on arthropod vectors of public health concern countrywide (Supplementary Materials). Knowledge, here, referred to what residents knew about arthropod vector biology, ecology, taxonomy and control practices. The dependent variables (knowledge items) were evaluated against the independent factors (e.g., district, age, gender and educational background) to quantify the responses using rating, nominal and ordinal scales following modifications from Buxton et al. [69]. 

Ethical clearance was attained from the Botswana International University of Science and Technology under a study permit from the Ministry of Environment, Natural Resources Conservation and Tourism (Botswana) (Permit **#**: ENT 8/36/4XXXXII(14). Preceding the data collection, consent was established with all respondents and details of the survey (aim, data collection and usage, issues of anonymity and confidentiality) were fully explained. The interviews were conducted in both Setswana (local language) and/or English, depending on the respondent’s language preference. Prior to the interviews, a pre-run (*N* = 30) was done to review and refine the questionnaire where necessary [55].

### 2.2. Data Analysis

Data were initially captured in Census and Survey Processing System software (CSPro 7.2) (United States Census Bureau) and subsequently analysed using IBM Statistical Package for Social Sciences (SPSS) version 26. Percentages and frequencies were used to report the data and variables that revealed statistical significances were separated at 95% confidence interval. The Pearson Chi-square test of association and Pearson’s correlation coefficient were used to test for the interaction effect between dependent and independent variables. 

## 3. Results

The gender of the respondents was 50.8% female and 49.2% male. The majority of the respondents were single (77.9%), literate (96.5%) and aged between 20–29 years (54.3%) (Table 1). Most of the respondents were located in semi-urban areas (46.5%), rural (33.5%), urban (19.3%) and very few were situated in peri-urban settlements (0.8%). The main sources of income were formal employment (32.1%), student allowance (31.7%) and small-scale self-employment (10.9%). Relatively few respondents obtained income as either assisted by family members (7.6%) or as large-scale entrepreneurs (4.8%), farming (4.7%), pension fund beneficiaries (1.4%) or poverty alleviation schemes (0.5%). Only 5.2% of respondents had no source of income with 0.9% citing other unspecified income sources (Table 1). The level of education was mostly tertiary (44.8%), upper secondary (Botswana Government Certificate of Secondary Education; BGCSE) (27.4%), with a few respondents having qualifications in lower secondary (Botswana Government Junior Certificate; BGJC) (11.9%), vocational training (10.8%) or primary (Primary School Leaving Examination; PSLE) (3.5%) training. Only 1.5% of respondents had no formal education training (Table 1). 

The majority of the respondents reported that arthropod vectors potentially transmit diseases to humans (92.5%), livestock (86.1%) and wildlife (72.3%) and this knowledge was significantly associated with the districts (*p* < 0.001) irrespective of the specific type of vertebrate host affected. The respondents from the North-West district were more knowledgeable on arthropod vectors transmitting diseases to hosts while Kgatleng exhibited the least knowledge. Amongst the common arthropod vectors, most of the respondents had prior knowledge of flies (99.7%), cockroaches (99.6%), mosquitoes (99.6%), ticks (99.1%), fleas (83.6%), lice (79.5%) and bedbugs (70.8%). The knowledge of cockroaches, here, was not significantly associated with districts (χ^2^ = 10.974, df = 10, *p* = 0.360). The knowledge of the arthropod vector was significantly linked to whether it transmitted diseases to humans (χ^2^ = 13.393, df = 4, *p* = 0.010) or livestock (χ^2^ = 20.088, df = 4, *p* < 0.001) but not in wildlife species (χ^2^ = 8.905, df = 4, *p* = 0.064). Moreover, there was a significant association between the respondents’ level of education and knowledge of arthropod vectors transmitting diseases to livestock (χ^2^ = 37.770, df = 10, *p* < 0.001) and wildlife (χ^2^ = 23.314, df = 10, *p* = 0.010) but not humans (χ^2^ = 10.643, df = 10, *p* = 0.386). Most of the respondents knew that there were different species of flies (82.2%), mosquitoes (71.9%) and cockroaches (61.7%) whilst the majority in plurality did not know if there were different species of bedbugs (46.6%), lice (39.4%) and fleas (38.6%). 

The arthropod vectors physically seen (in a lifetime) by the respondents were reported 99.5%, 99.4%, 99.1%, 97.1%, 72.1%, 51.5% and 48.4% for cockroaches, mosquitoes, flies, ticks, fleas, lice and bedbugs, respectively. Age was also significantly associated with respondents who had previously seen the lice (χ^2^ = 199.600, df = 10, *p* < 0.001), bedbugs (χ^2^ = 120.603, df = 10, *p* < 0.001) and fleas (χ^2^ = 49.422, df = 15, *p* < 0.001) than those who had seen cockroaches (χ^2^ = 7.729, df = 10, *p* = 0.655), ticks (χ^2^ = 11.696, df = 10, *p* = 0.306), flies (χ^2^ = 8.423, df = 15, *p* = 0.906) or mosquitoes (χ^2^ = 2.005, df = 10, *p* = 0.996). Middle-aged adult respondents from 30–39 years and above (as opposed to the youth of ≤ 29 years) had the highest number of responses, exhibiting awareness of lice, bedbugs and fleas. Only 48.4% had seen bedbugs, while 45.9% had not and 5.7% were not sure. As such, familiarity with an arthropod vector was significantly associated with having previously seen cockroaches (χ^2^ = 17.853, df = 4, *p* < 0.001), lice (χ^2^ = 297.535, df = 4, *p* < 0.001), ticks (χ^2^ = 9.924, df = 4, *p* = 0.042), bedbugs (χ^2^ = 53.570, df = 4, *p* < 0.001) or fleas (χ^2^ = 26.470, df = 6, *p* < 0.001) but not flies (χ^2^ = 1.430, df = 6, *p* = 0.964) or mosquitoes (χ^2^ = 4.287, df = 4, *p* = 0.369). 

**Figure 2:**
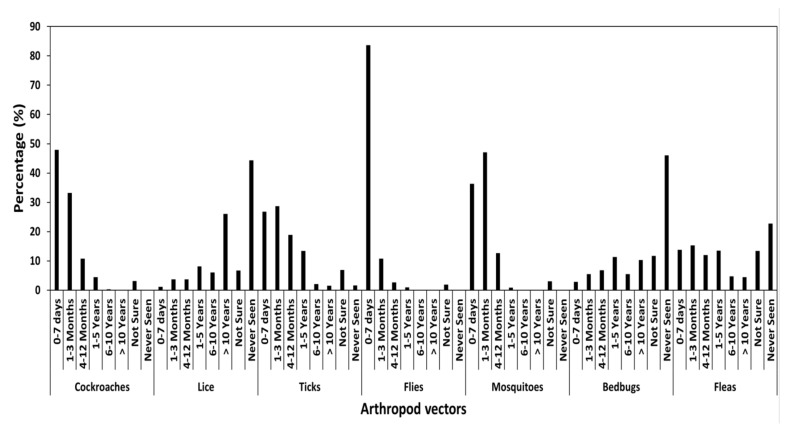
Summary of responses (%) of respondents who reported seeing arthropod vectors over different timescales.

The majority of those who had last seen arthropod vectors in the previous 0–7 days were 83.7% and 47.9% for flies and cockroaches, respectively (Figure 2), whereas the majority of those who last saw them between one to three months were 47.1% and 28.7% for mosquitoes and ticks, respectively (Figure 2). Most of the respondents who had never seen the arthropod vectors were 46.0%, 44.3% and 22.8% for bedbugs, lice and fleas, respectively (Figure 2). 

Most arthropod vectors were reportedly identified morphologically by their shape for cockroaches (66.1%), ticks (61.0%), flies (48.9%), mosquitoes (40.0%) and fleas (38.7%) while 51.1% and 47.9% did not know how to identify bedbugs and lice, respectively. Arthropod pest infestation was experienced by 84.0%, 74.5%, 73.0%, 48.1%, 34.8%, 17.7% and 13.6% for mosquitoes, cockroaches, bedbugs, ticks, fleas, bedbugs and lice, respectively. The majority of those who did not experience infestation within their households were 79.0% (lice), 70.1% (bedbugs), 57.8% (fleas), 50.0% (ticks), 26.1% (cockroaches), 24.4% (flies) and 15.4% for mosquitoes. There was no significant association of urban pests with the type of location, i.e., for cockroaches (χ^2^ = 7.838, df = 6, *p* = 0.250) and flies (χ^2^ = 7.349, df = 6, *p* = 0. 290). Ticks (*p* < 0.001) mosquitoes (*p* = 0.014) and lice (*p* < 0.001) reported a significant link of pest infestation with the type of location. Thus, the highest infestation for both ticks and mosquitoes was displayed in semi-urban areas while for lice in rural areas. The infestation of mosquitoes was significantly associated with the respondents’ district (χ^2^ = 61.486, df = 20, *p* < 0.001). Malaria endemic districts (e.g., North-West and Chobe) had the highest mosquito infestation reports, as opposed to most non-malaria endemic areas (e.g., Southern, South-East and Kgatleng). However, other non-endemic malaria areas (e.g., Ghanzi, Kgalagadi and Kweneng) reported high mosquito infestations, similar to those reported in malaria endemic districts.

**Figure 3:**
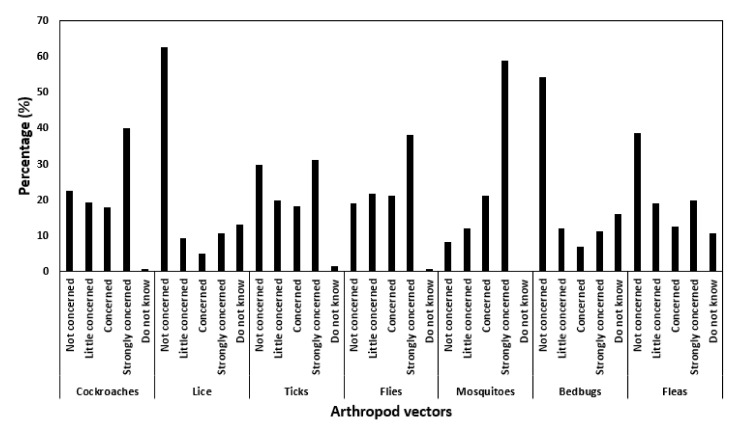
Summary of respondents’ perceptions (%) on vector arthropods rated as; not concerned, little concerned, concerned, strongly concerned, and did not know.

Whilst many factors can attract arthropod vectors, most respondents associated host availability with ticks (80.3%) and fleas (54.1%), whereas pit latrines, food waste and stagnant waters were linked with cockroaches (38.8%), flies (35.0%) and mosquitoes (32.0%), respectively. Most respondents did not know what attracted bedbugs (58.7%) and lice (43.8%). Similarly, most respondents were also strongly concerned about the burden of mosquitoes (58.9%), cockroaches (39.8%), flies (38.0%), ticks (31.1%), fleas (19.6%), bedbugs (11.1%) and lice (10.5%). However, some were not concerned about the burden of lice (62.6%), bedbugs (54.3%), fleas (38.6%), ticks (29.6%), cockroaches (22.5%), flies (19.0%) and mosquitoes (8.1%) (Figure 3). 

The majority of the respondents who were certain that arthropod vectors were responsible for the transmission of pathogens and parasites were 96.9%, 75.3%, 65.9% and 60.7% for mosquitoes, flies, ticks and cockroaches, respectively. However, most respondents were not sure if bedbugs (61.3%), lice (53.1%) and fleas (47.2%) played any role in disease transmission. The majority of the respondents did not know any specific disease transmitted by bedbugs (76.8%), lice (74.3%), fleas (72.0%), ticks (54.1%), cockroaches (52.3%) and flies (44.0%) while malaria (93.0%) was mostly known to be caused by the parasites carried by mosquitoes. The lack of knowledge about other diseases transmitted by mosquitoes was not significantly linked to the respondent’ district (χ^2^ = 70.806, df = 60, *p* = 0.160) indicating that knowledge paucity was countrywide. However, there was a significant association of knowledge and district on diseases transmitted by cockroaches, lice, flies, ticks, bedbugs and fleas (*p* < 0.001).

**Figure 4:**
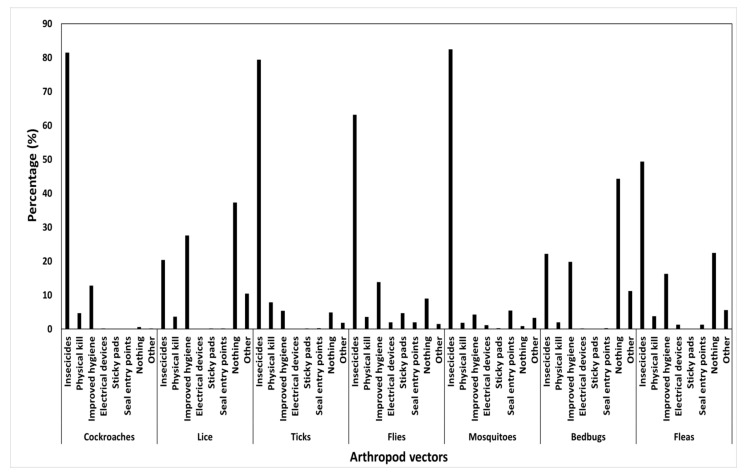
Summary responses (%) showing different ways in which arthropod vectors are controlled within households.

The arthropod vectors that were mostly thought to be abundant in summer were mosquitoes (88.0%), cockroaches (68.6%) and flies (63.7%), while ticks (39.2%) were thought to be abundant all year round. Most of the respondents were not aware of the season of abundance for bedbugs, lice and fleas at 63.7%, 57.2% and 45.3%, respectively. Ticks (65.0%), fleas (39.3%) and flies (35.0%) were reported to be active all day while mosquitoes and cockroaches were reportedly nocturnal, with no knowledge of the activity time for bedbugs (49.0%) and lice (46.6). Synthetic insecticides were used by the majority of the respondents for controlling mosquitoes (82.5%), cockroaches (81.5%), ticks (79.4%), flies (63.2%) and fleas (49.4%) while no control measures were effected against bedbugs (44.3%) and the lice (37.3%) (Figure 4). There was a significant interaction effect on the utilisation of synthetic insecticides against mosquitoes across districts (χ^2^ = 264.308, df = 70, *p* < 0.001). Thus, a malaria endemic district (North-West) exhibited the highest usage of synthetic insecticidal usage while the non-malaria endemic (Kgatleng) showed the least. The control of cockroaches (*p* = 0.954), lice (*p* = 0.780), ticks (*p* = 0.985), flies (*p* = 0.203), mosquitoes (*p* = 0.874), bedbugs (*p* = 0.400) and fleas (*p* = 0.976) were not significantly associated with the literacy status of respondents. The type of location was not significantly linked to the control of cockroaches (*p* = 0.855), lice (*p* = 0.383), flies, mosquitoes (*p* = 0.328), bedbugs (*p* = 0.593) and fleas (*p* = 0.480). However, ticks had a significant interaction, reporting the highest utilisation of synthetic insecticides in semi-urban locations with the least in peri-urban (χ^2^ = 31.828, df = 18, *p* = 0.023).

**Figure 5:**
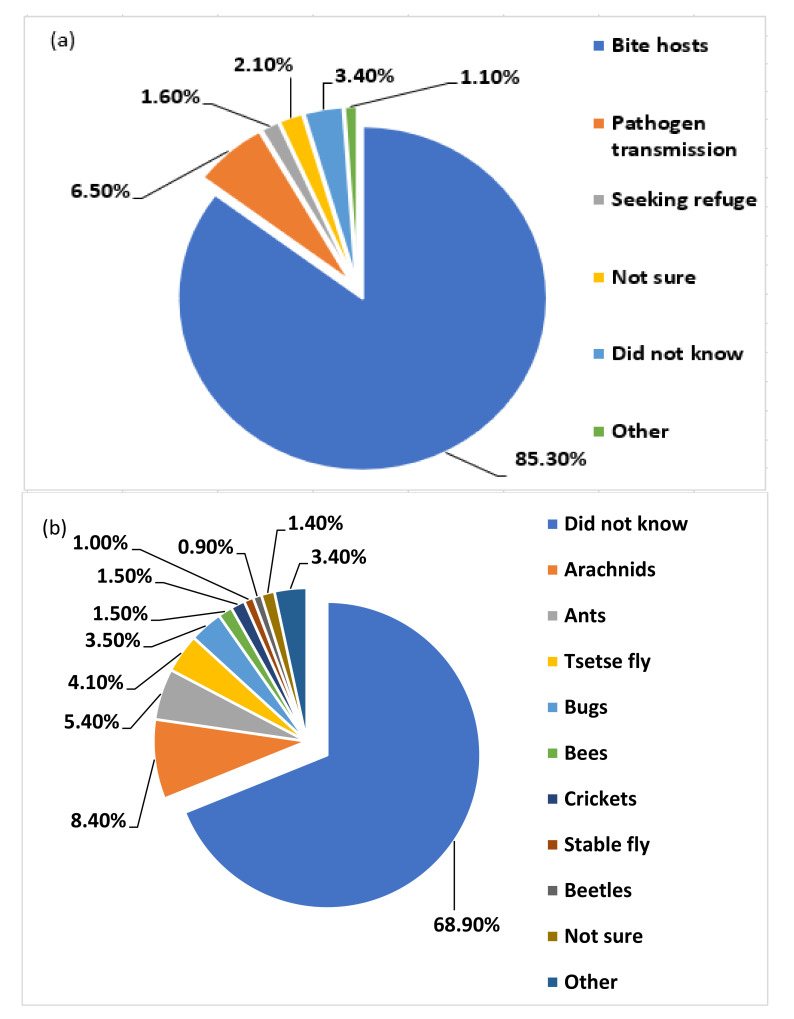
Summary responses (%) of respondents on (**a**) the urge for arthropod vectors to feed/ infest hosts and (**b**) some of arthropod vectors thought to be of potential health threats in Botswana.

Most respondents reported the urge to feed as the main reason for arthropod vectors to bite hosts (85.3%) as opposed to pathogen/ parasite transmission (6.5%) or seeking refuge (1.6%). Only 2.1% were not sure while 3.4% did not know (Figure 5a). Whilst the majority of the respondents did not know other arthropod vectors (68.9%) that could be problematic in the country, arachnids (e.g., mites) (8.4%), ants (5.4%), tsetse fly (4.1%), bugs (3.5%), bees (1.5%), crickets (1.5%), stable fly (1.0%) and beetles (0.9%) were thought to be somehow posing health threats in Botswana. Only 1.4% were not sure or did not specify any problematic arthropod vector (3.4%) (Figure 5b). However, respondent’s age was significantly associated with the knowledge of other vector arthropods that could be linked with health risks in the country (χ^2^ = 50.991, df = 50, *p* = 0.036).

## 4. Discussion

This study revealed that the communities in Botswana were aware of potentially pathogenic-incriminated arthropod vectors of health concern, including mosquitoes, cockroaches, lice, ticks, flies, bedbugs, fleas and other related problematic organisms. The study showed a descending level of concern from mosquitoes (highest), cockroaches, flies, ticks, fleas, bedbugs and lice (lowest). Our results showed that arthropod vector control was predominantly using synthetic pesticides. The study also showed that the infestation by urban arthropod pests (e.g., cockroaches, flies) was similar across districts. Generally, community knowledge on arthropod vectors was limited (e.g., species identity, parasites/pathogens and associated diseases transmitted and control practices). Similarly, respondents’ knowledge on mosquito-borne infections was skewed towards only malaria, regardless of the district’s malaria endemicity status. 

Mosquitoes, ticks and their burdens were widely explored in the region because of their significant local contribution to human malaria [18,72], livestock (e.g., heartwater) [22] as well as zoonotic diseases [73]. Despite the knowledge of diversity of arthropod vector groups explored in this study, malaria was the only prominent mosquito-borne infectious disease known to most of the respondents’ households. Mosquito infestation was mainly reported in malaria endemic districts as opposed to the non-malaria districts. However, some non-malaria districts (e.g., Ghanzi, Kgalagadi and Kweneng) reported high mosquito infestations. This could be a case of other vector mosquito species such as *Culex* and *Aedes*. Recent evidence also suggests that cattle dung eutrophication is linked with mosquito abundance [62], likely explaining the increased vectors in these high livestock areas. Moreover, in recent years, malaria sporadic cases were increasingly reported in non-malaria districts countrywide [74]. This is a worrying scenario that needs exploration regarding transmission dynamics of vector, pathogen, host and environmental factor interaction. The respondents’ level of education was significantly linked to their knowledge of arthropod vectors transmitting diseases to livestock and wildlife but not humans. This result is in consonance with the large investments by the government in livestock [75] and wildlife touristic enterprises [76] and related information thereof. However, this skewed knowledge may come at a large cost associated with the lack of knowledge of arthropod vectors that directly affect human health. Future investment should focus on closing this gap that likely highlights potentially unbalanced emphasis across various sectors of livelihood development. Similarly, we observed disparity in knowledge gaps amongst the districts. For instance, the Kgatleng District displayed limited knowledge on arthropod vectors compared to the North-West district. We speculate, with caveats, that this could be associated with whether the district is malarious or not. The North-West is a malarious district and because of the chronic malaria cases in the district, it is highly likely that respondents from this district will be aware of mosquito vector species, and by extension, other pathogenic-incriminated arthropod vectors of health concern. This may also mean that vector information dissemination may likely be prioritised in high-risk regions as opposed to the less affected area, creating a knowledge transfer imbalance and thus gaps in some localities. Owing to this limited and skewed knowledge in several aspects of arthropod vector bio-ecology (e.g., identification, control practices), stakeholders are urged to harmonise and strengthen awareness campaigns and to use area-wide approaches amongst communities countrywide.

The control of arthropod vectors was mainly reported to be synthetic chemical based at the household level. Similarly, at the national level (e.g., mosquito vectors), *Anopheles* species were mainly targeted and controlled through synthetic insecticidal approaches in malaria endemic districts of the country, at least since 1950s [17,64,70]. Although insecticides are most effective in the control of vectors, they are currently conferring resistance to targeted vector pest species locally [17], regionally [77,78] and worldwide [79,80]. This challenge may be addressed by promoting other complementary control strategies (e.g., utilising indigenous knowledge-based solutions/naturally inherent ecosystem services) other than solemnly relying on synthetic chemicals [81,82,83]. However, control strategies may be unique across the specific demands of diverse bio-physical structures.

Majority of the respondents were strongly concerned about mosquitoes, cockroaches, flies and ticks in the country but less concerned about fleas, bedbugs and lice. The infestation of urban arthropod pests (cockroaches and flies) was similar across districts and moreover, were seen within a week (0–7 days) prior to the survey. Mosquitoes and ticks were only seen within 1–3 months. The reason could be that the survey was conducted during the austral winter when arthropod vector populations and abundance were likely reduced [84]. Considering these dynamics, mosquitoes, cockroaches, flies, ticks and bedbugs may need to be prioritised as health risks to the communities across the country given their cosmopolitan presence and intensity, frequent activity and their abundance across seasons, albeit mainly in summer. Little has been done on cockroaches and flies as important transmitters of disease although they were reported as of major concern countrywide [85]. Cockroaches are mainly involved in the mechanical, transmission of parasites (e.g., *Ascaris, Capillaria, Trichuris, Capillaria* and *Toxocara,* hook worms) that infect humans [86]. Similarly, flies such as *Musca domestica* are mechanical vectors of disease-causing pathogens (e.g., fungi, bacteria, viruses and parasites) to humans and domesticated livestock [87]. Thus, local flies (e.g., stable, horse, house and blow flies) may be important arthropod vectors of medical and veterinary importance in triggering and exacerbating secondary infections directly or indirectly. Although mosquito abundance and refugia hotspots may be influenced by climatic and anthropogenic activities (e.g., degraded habitats) [69,88,89], this study further reported that pit latrines and food wastes were likely the major attractants to cockroaches and flies in homesteads. Therefore, it remains essential to assess the pathogenicity of urban pests across rural-urban landscapes.

The majority of the respondents considered lice, bedbugs and fleas as of least health concern nationwide. Although these arthropod vectors have a long history and earliest recorded evidence of existence in Africa, they persist and continually live in close association with humans [90]. It is likely that infestation by these species is declining mainly due to improved housing quality, hygiene and living conditions in modern households [91]. In Botswana, no studies to our knowledge have explored the prevalence, diversity, distribution, and public health burdens associated with lice, bedbugs and fleas. Despite reports of these vectors in the country from this study, the findings should be interpreted with caution given the knowledge gaps of various disease-causing arthropods across the communities. Therefore, field surveillance of these vectors may be necessary countrywide to ascertain the claim of their prevalence, abundance, bio-ecology (e.g., life history traits and breeding sites) and associated parasites/pathogens as their infestation significantly varied across districts. 

Temporal activity of arthropod vectors has been reported regionally and worldwide [92,93]. Thus, current global change has a bearing on favouring vector success and proliferation, pathogen/parasite virulence and disease transmission dynamics in shifting climatic environments [94,95,96]. Whilst the regional climate is changing [97], species range expansion, re-distribution and invasion in novel environments are inevitable [98]. To a greater extent, mosquitoes are the only vectors that have to date, been given priority in Botswana [15,17] and indeed the region (see [99]). This has created an intervention and knowledge void in other areas of the country even in areas where malaria transmitting vector mosquito species are emerging [15,16,18]. Consequently, spatio-temporal monitoring of all arthropod vectors highlighted by the study is crucial to establish baseline data for evaluating population dynamics and potential early warning systems in time and space.

## 5. Conclusions

Whilst the country’s efforts are more mosquito- and malaria-centric, there are other significant arthropod vectors of community/ health concern, including cockroaches, flies, ticks, fleas, bedbugs and lice. These potentially transmit pathogens of debilitating human, livestock and wildlife diseases that may affect community socio-economic activities. This study showed that while communities were aware of arthropod vectors, the knowledge on their biology, ecology and control options was limited. The infestation by urban pests such as mosquitoes, cockroaches, flies and ticks were of strong concern by the majority, thus necessitating investment in effective and sustainable control methods in reducing their impacts on public health. Furthermore, apart from mosquitoes and malaria, results showed a concerning skewed knowledge on vectors of wildlife and livestock diseases, seemingly at the cost of vectors of human disease, that warrants further public education campaigns. Whilst the prevalence of arthropod vectors has been reported in this study, it remains crucial to physically monitor species’ spatio-temporal distribution and abundance through strict surveillance studies as a step towards managing emerging and re-emerging infectious diseases to inform early warning systems. The findings of this study are important to the communities and policy makers in public health sectors for improved community health.

## Figures and Tables

**Table 1:** A summary of results on the socio-demographic characteristics of respondents across the ten major districts of Botswana (*N* = 1048). The valid percentage (i.e., whereby the total number of responses did not include the missing values) was used for the determination of reported proportions.

Variables	Category	Number of Respondents (*N* = 1048)	Proportion (%)
Gender	Male	506	48.3
	Female	532	50.8
	Prefer not to say	10	1.0
Marital Status	Single (never married)	816	77.9
	Married	168	16
	Divorced	13	1.2
	Widowed	11	1.1
	Staying together	22	2.1
	Prefer not to say	17	1.6
Age (years)	16–19	77	7.4
	20–29	567	54.3
	30–39	187	17.9
	40–49	121	11.6
	50–59	54	5.2
	≥60	38	3.6
Literacy	Literate	1000	96.5
	Illiterate	17	1.6
	Not sure	19	1.8
Education	None	16	1.5
	Primary	36	3.5
	Junior Certificate	124	11.9
	Form 4–5 (BGCSE)	286	27.4
	Vocational	113	10.8
	Tertiary	467	44.8
Location	Rural	350	33.5
	Semi-urban	485	46.5
	Urban	201	19.3
	Peri-urban	8	0.8
Source of income	Employee	339	32.4
	Entrepreneur	50	4.8
	Self employed	114	10.9
	Student allowance	332	31.7
	Farmer	49	4.7
	Pension fund	15	1.4
	Poverty alleviation fund	5	0.5
	Family member(s)	79	7.6
	Nothing	54	5.2
	Other	9	0.9

## Data Availability

Although we did not acquire consent to share data obtained from the questionnaire, the datasets used and/or analysed during the current study may be available from the corresponding author on reasonable request.

## Supplementary Materials

The following are available online at https://www.mdpi.com/article/10.3390/ijerph181910556/s1, Household Questionnaire.
