# Supplementary material for: A Survey of Potentially Pathogenic-Incriminated Arthropod Vectors of Health Concern in Botswana
Source: Int J Environ Res Public Health. 2021 Oct 8;18(19):10556. doi: 10.3390/ijerph181910556 (PMC8508065; doi:10.3390/ijerph181910556)
Supplement: Supplementary file 1 [file ijerph-18-10556-s001.zip › ijerph-1340519-supplementary.pdf]

A survey of potentially pathogenic-incriminated arthropod vectors of health concern in Botswana

Survey summary

Arthropods play a critical role as disease vectors causing morbidity and mortality globally. The current global change projections are likely to proliferate vectors and associated pathogens, exacerbating arthropod-borne infection risks. Common vectors of medical importance include; cockroaches, lice, bedbugs, fleas, ticks, flies and mosquitoes. This survey is a baseline scoping of potential arthropod vectors of health concern in Botswana. The main aim is to ascertain spatio-temporal patterns in knowledge and perceptions of these potential vector arthropods by the public. This will bridge knowledge gaps for improvement in health, well-being and livelihoods across the country. The survey is voluntary and can be completed by individuals above ≥16 years of age.

Household Questionnaire

Interviewer ID:

Date: ____________ / _______________/ 2020

Respondent Name: **Surname:**

1. Do you agree to participate in this survey?

Yes=1 No=2

**2. Do you know if any arthropods carry/ cause diseases to;**	Yes=1<br>No=2<br>Not sure=3
(i) Humans?	
(ii) Livestock?	
(iii) Wildlife?	

BIUST: Department of Biological Sciences and Biotechnology

Questionnaire Code:

Questionnaire number	Interviewer Initials

2020

Arthropod<br><br>vectors	**3. Do you know**<br><br>Yes=1<br>No=2<br>Not sure=3	**4. Are there different types of this vector?**<br><br>Yes=1<br>No=2<br>Not sure=3<br>I don't Know=4	**5. Have you seen the vector?**<br><br>Yes=1<br>No=2<br>Not sure=3	**6. What feature(s) do you use to identify the vector?**<br><br>Shape=1<br>Movement=2<br>Colour=3<br>Sound=4<br>Bites=5<br>I don't know=6<br>Other=7	**7. Last time seen?**<br><br>0-7days=1<br>1-3months=2<br>4-12months=3<br>1-5yrs=4<br>6-10yrs=5<br>≥10years=6<br>Not sure=7<br>Never seen it=8	**8. Have you ever experienced infestation in your household?**<br><br>Yes=1<br>No=2<br>Not sure=3	**9.What attracts this vector in homesteads?**<br>Pit latrines=1<br>Sewage systems=2<br>Rubbish=3<br>Food=4<br>Warm places=5<br>Suitable refuge space=6<br>Hosts=7<br>I don't know=8<br>Other=9	**10. How concerned are you about its burden?**<br><br>Not concerned=1<br>Little Concerned=2<br>Concerned=3<br>Strongly Concerned=4<br>I don't know=5	**11.Do they transmission pathogens causing diseases?**<br><br>Yes=1<br>No=2<br>Not sure=3	**12. Name of diseases transmitted/ caused**	**13. Season of abundance.**<br><br>Summer=1 (Nov-Jan)<br>Autumn=2 (Feb-Apr)<br>Winter=3 (May- Jul)<br>Spring=4 (Aug-Oct)<br>All year round=5<br>I don't know=6	**14. When are vectors more active?**<br><br>Morning=1<br>Afternoon=2<br>Evening=3<br>Night=4<br>All day=5<br>Not Sure=6<br>I don't know=7	**15. How do you control the vectors?**<br><br>Insecticides=1<br>Physical kill=2<br>Improved hygiene=3<br>Electrical devices=4<br>Sticky pads=5<br>Seal/ close entry points=6<br>Nothing=7<br>Other=8
(i)<br><br>Cockroaches													
(ii)<br><br>Lice													
(iii)<br><br>Ticks													
(iv)<br><br>Flies													
(v)<br><br>Mosquitoes													
(vi)<br><br>Bedbugs													
(vii)<br><br>Fleas													

16. Why do you think arthropod vectors bite?

To transmit parasites=1 For feeding (food)=2 Seek refuge=3 Not sure=4 I don't know=5 Other=6__________________

17. Which other arthropod vector(s) do you think are of public health concern in Botswana?

____

18. In which district do you live?

North-West=1 North-East=2 South-East=3 Southern=4 Ghanzi=5 Chobe=6 Kgatleng=7 Kweneng=8 Central=9 Kgalagadi=10

19. Describe your locality (area of residence).

Rural=1 Semi-urban=2 Urban=3 Peri-urban=4

20. What is your source of income?

Employee=1 Entrepreneur=2 Self-employed=3 Student allowance=4 Farmer=5 Pension fund=6 Government poverty alleviation funds=7 Nothing=8 Family=9 Other=10________________

21. Marital status

Single=1 Married=2 Divorced=3 Widowed=4 Staying together=5 Prefer not to say=6

22. Are you literate?

Yes=1 No=2 Prefer not to say=3

23. What is your highest level of education?

None=1 Primary=2 BGJC (form 3)=3 BGCSE (form 5)=4 Vocational=5 Tertiary=6

24. Age

16- 19=1 20- 29=2 30- 39=3 40- 49=4 50- 59=5 60+ years=6

25. Gender

Female=1 Male=2 Prefer not to say=3
